# Population-based Survey of Invasive Bacterial Diseases, Greenland, 1995–2004

**DOI:** 10.3201/eid1401.071240

**Published:** 2008-01

**Authors:** Annette Meyer, Karin Ladefoged, Peter Poulsen, Anders Koch

**Affiliations:** * Statens Serum Institut, Copenhagen, Denmark; † Dronning Ingrids Hospital, Nuuk, Greenland

**Keywords:** epidemiology, bacterial infections, sepsis, meningitis, Streptococcus pneumoniae, Inuits, Greenland, Arctic regions, dispatch

## Abstract

Invasive bacterial disease occurs frequently among native populations in the Arctic. Although a variety of bacteria are involved in invasive bacterial disease in Greenland, *Streptococcus pneumoniae*, *Escherichia coli*, *Staphylococcus aureus*, and other staphylococci are responsible for most cases (69%); incidence varies according to region and ethnicity.

The incidence of invasive disease caused by *Streptococcus pneumoniae* and several other bacteria is markedly higher in the Inuit populations of Alaska, Canada, and Greenland than in the non-Inuit populations of the same areas (*1*–*3*). The clinical extent of invasive disease caused by *S. pneumoniae* is severe; sequelae and case-fatality rates are almost 4× higher in native than in nonnative populations (*1*,*4*). As living conditions in Arctic populations in many ways are comparable, an international, cooperative, population-based surveillance for invasive diseases was established in 1999 (*5*). The International Circumpolar Surveillance (ICS) network registers invasive disease caused by *S. pneumoniae*, *Haemophilus influenzae*, *Neisseria meningitidis*, group A streptococcus, and group B streptococcus in Alaska, Canada, Greenland, Iceland, northern Norway, Finland, and Sweden (*5*).

In Greenland, case data for this network are reported from the treating doctors to the chief medical officer of Greenland, who in turn reports to the ICS. However, the full picture of invasive bacterial disease in Greenland and the relative extent of disease caused by the 5 bacteria on which the ICS focuses have been unknown. Using laboratory data for a 10-year period in Greenland, we determined microbiologic causes of all invasive bacterial disease cases, microbial-specific trends in incidence over time, and variation in incidence by ethnicity and place of residency.

## The Study

Greenland is inhabited by 56,000 persons. One fourth of the population lives in the capital, Nuuk, while the rest live in 16 towns and several settlements (the districts) scattered around the island. Most persons (86%) are native Greenlanders; the rest are Caucasians, mostly Danes.

The laboratory at Queen Ingrids Hospital in Nuuk is the only microbiologic laboratory in Greenland. From laboratory files, we identified all bacterial isolates from invasive disease cases reported from January 1, 1995, though December 31, 2004. Demographic information about patients was obtained from the Civil Registration System of Greenland (*6*). Patients were categorized as Greenlanders if born in Greenland. Population data for incidence calculations were obtained from Statistics Greenland (*7*). The study was approved by the Commission for Scientific Research in Greenland and reported to the Danish Data Protection Agency.

The laboratory used standard microbiologic methods. Until 1999, positive blood cultures were detected by using manual readings of blood culture bottles from Statens Serum Institut, Copenhagen, Denmark; beginning in 1999, the Bactec/Alert system (Organon Teknika, Turnhout, Belgium) was used. In this system, 28–30 mL of blood is injected directly into aerobic and anaerobic blood culture bottles. Positive samples are characterized by Gram stain and cultured on blood agar supplemented with 5% defibrinated horse blood. Antigen/antibody tests are carried out for *S. pneumoniae*, *N. meningitidis*, and *H. influenzae* (the last 2 only on cerebrospinal fluid). Serotyping and group typing of *S. pneumoniae and H. influenzae* (Quellung method) and *N. meningitidis* (latex agglutination method) are performed at Statens Serum Institut.

In total, 281 unique bacterial isolates from 254 episodes of invasive disease among 242 patients were identified: 72% of isolates in blood, 18% in cerebrospinal fluid, and 10% in other samples. Of the 254 episodes, 47% occurred among patients from the districts and 53% in patients from Nuuk. In 53% of episodes among patients from the districts, the first microbiologic sample was taken after patient transfer to Nuuk. There were no differences in age, sex, ethnicity, underlying conditions, or mortality rates between district and Nuuk patients, but Greenlandic patients were younger (median 46 years, 25%–75% quartiles 17–59 years) than Danish patients (median 57 years, 25%–75% quartiles 44–62 years) (p = 0.07).

**Figure 1:**
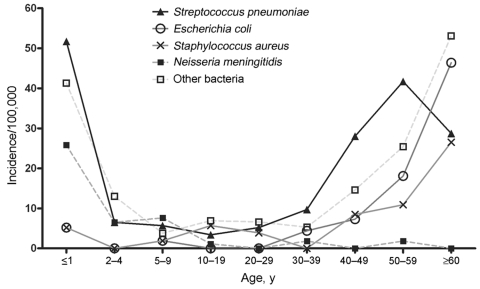
Incidence by age, invasive bacterial disease, Greenland, 1995–2004.

Twenty different bacterial species or groups were identified (22 isolates could not be characterized by species); the most numerous were *S. pneumoniae,* staphylococci, and *E. coli* (Table). For most bacteria, the invasive disease incidence varied by age in a U-shaped fashion (Figure 1). Most *N. meningitidis* cases (73%) occurred in children <10 years of age, the 4 cases of *H. influenzae* group B infection occurred in children <1 year of age, and the youngest case-patient with GBS was 12 years of age.

**Table:** Characteristics of 281 bacterial isolates and 242 case-patients from 254 episodes of invasive bacterial infection, Greenland, 1995–2004

Bacterial class	No. isolates	Median age, y (range)	Overall incidence*	Incidence* by sex			Incidence* by ethnicity (place of birth)			Incidence* by region (place of living)	
				M (n = 129)	F (n = 125)		Greenland (n = 235)	Denmark/other (n = 18)		Nuuk (n = 134)	Districts (n = 120)
*Streptococcus* spp.											
*S. pneumoniae†*	92	46 (0–76)	16.4	15.7	17.2		17.6	8.0		39.9	8.7
Group A streptococci	5	58 (37–76)	0.9	1.3	0.4		1.0	0		0.7	0.9
Group B streptococci	5	43 (12–70)	0.9	1.0	0.8		1.0	0		2.9	0.2
Other‡	10	38 (6–67)	1.8	2.7	0.8		1.8	1.6		5.1	0.7
*Staphylococcus* spp.											
*S. aureus*§	35	50 (0–90)	6.2	4.7	8.0		6.5	4.8		13.8	3.8
Other¶	22	41 (0–70)	3.9	4.7	3.1		4.1	3.2		7.3	2.8
*Enterococcus faecalis*	8	45 (0–72)	1.4	2.0	0.8		1.4	1.6		2.9	0.9
*Neisseria meningitidis#*	15	5 (0–54)	2.7	2.3	3.1		3.0	0		5.8	1.6
*Moraxella catarrhalis*	2	18 (0–36)	0.4	0	0.8		0.4	0		0.7	0.2
*Haemophilus influenzae*											
Type b	4	0 (0–0)	0.7	0.7	0.8		0.8	0		0.7	0.7
Non-b	10	22 (0–71)	1.8	1.3	2.3		2.0	0		2.9	1.4
*Pseudomonas aeruginosa*	2	45 (36–55)	0.4	0.7	0		0.4	0		1.5	0
Enterobacteriaceae spp.											
*Escherichia coli*	44	58 (0–84)	7.8	7.7	8.0		7.9	8.0		14.5	5.7
*Klebsiella* *pneumoniae*	6	67 (22–74)	1.1	1.0	1.1		1.0	1.6		2.9	0.5
*Salmonella* spp.	5	42 (12–51)	0.9	1.0	0.8		0.6	3.2		2.2	0.5
*Enterobacter cloacae*	1	72	0.2	0.3	0		0.2	0		0	0.2
Other											
Gram-positive cocci**	5	46 (4–65)	0.9	0.7	1.1		1.0	0		1.5	0.7
Gram-positive rods††	7	58 (13–72)	1.2	1.3	1.1		1.4	0		2.2	0.9
Gram-negative rods‡‡	3	54 (13–72)	0.5	0	1.1		0.6	0		0.7	0.5
Total	281	47 (0–90)	50.0	49.0	51.1		52.9	32.1		108.1	31.1

*Per 100,000 population. †Most numerous serotypes were 12F (9) and 22F (5); 41 isolates not were serotyped. Serotype distribution for 1996–2002 described in detail in (*1*). ‡Other hemolytic and nonhemolytic streptococci. §None of the *S. aureus* isolates were methicillin-resistant strains. ¶*S. epidermidis* (6) and other non–*S. aureus* staphylococci. #Two isolates in group B, 4 in group C, and 9 not grouped. **S. mitis (1) and unspecified cocci. ††*Bacillus* sp. (1), corynebacterium (1), *Clostridium perfringens* (2), *Listeria monocytogenes* (1), and *Propionebacterium* sp. (1). ‡‡*Bacteroides* sp. (1) and unspecified rods.

Incidence of invasive bacterial disease did not differ according to patient sex but did differ markedly according to ethnicity and region. The age-adjusted rate ratio was 1.8 (95% confidence interval [CI] 1.1–2.9) for Greenlanders compared with Danes and 3.5 (95% CI 2.7–4.4) for persons living in Nuuk compared with persons living in the districts, a rate ratio observed for almost all bacterial species.

**Figure 2:**
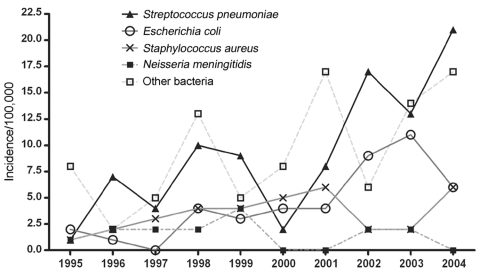
Incidence by year, invasive bacterial disease, Greenland, 1995–2004.

The overall incidence of invasive bacterial isolates increased during the study period from 17.9/100,000 in 1995 to 79/100,000 in 2004; the most marked increase occurred in 1997–1998, when incidence almost doubled. The increase occurred mainly in blood culture samples; cerebrospinal fluid samples remained constant over time. The increase in isolates from blood cultures occurred equally in Nuuk and in the districts and for most bacteria, although the incidence of *N. meningitidis* remained constant (Figure 2). *H. influenzae* group B isolates were not identified after 1998 (childhood vaccination was introduced in 1997); the 5 GAS isolates first appeared in 2001–2004.

## Conclusions

A variety of bacteria were found to be associated with invasive disease in Greenland. *S. pneumoniae* was the most commonly involved, confirming the importance of this pathogen in native populations in the Arctic (*1*–*3*). However, the second and third most frequent invasive bacteria were *E. coli* and *S. aureus*, a finding which, to our knowledge, had not been described before in Greenland. In contrast, the 5 bacteria included in the Circumpolar Surveillance System accounted for only 47% of microorganisms (*5*). Surveillance of these 5 bacteria is relevant, though, as they are all serious, potentially fatal diseases of infants or children and are associated with some form of preventive action (either vaccines or prophylaxis). *S. aureus* and *E. coli* are not amenable to simple specific public health actions, so surveillance for these infections is less useful. Evaluation of outcome and risk factors for *S. aureus* and *E. coli* in Arctic areas may lead to potential preventive or clinical care options.

Worldwide, microbiologic causes of bacteremia have shifted from gram-negative organisms being the most common in the 1970s to gram-positive organisms with coagulase-negative staphylococci, *S. aureus*, and enterococci being the most frequent in the last part of the 20th century (*8*). Likewise, we found gram-positive bacteria in 67% of invasive disease cases and gram-negative bacteria (mostly Enterobacteriaceae) in 33% and increasing incidence of both gram-positive and gram-negative bacteria over the study period.

The overall incidence of invasive bacterial disease was 3.5-fold higher among Nuuk patients than among district patients. Most likely this finding represents sampling bias; microorganisms sent from the districts are less likely to survive than samples taken in Nuuk, and district doctors may be less likely to submit samples than Nuuk doctors, given the longer time to receive test results. However, we cannot confirm this explanation because of lack of information on the total number of submitted samples. Furthermore, the 39% higher average income in Nuuk compared with the rest of Greenland makes a Nuuk/district invasive disease incidence rate ratio of 3.5:1 less likely (*7*). The difference suggests that the true invasive disease rates in Greenland may be much higher than those found in this study or reported to ICS. A clear recommendation from this study is to develop a more accessible, rapid, and reliable diagnostic system for districts outside of Nuuk.

The lower age-adjusted invasive bacterial disease incidence in Danes than Greenlanders may be explained by the “healthy worker effect.” Danes in general come to Greenland to work and belong to higher social classes and live in better housing conditions than the general population of Greenland, factors that may reduce the risk for infection (*9*). Hence, although genetic factors may account for some of the difference, the difference is probably confounded by environmental and social factors.

The increase in invasive bacterial isolates during the study period most likely represents changes in submission or detection practices. The increase occurred almost exclusively in blood cultures, and a new blood culture detection system was introduced in 1999. Similar findings have been observed in Europe (*10*). These data may lead to improved surveillance of invasive bacterial disease in Greenland.
